# Surface dose measurements with commonly used detectors: a consistent thickness correction method

**DOI:** 10.1120/jacmp.v16i5.5572

**Published:** 2015-09-08

**Authors:** Tatsiana A. Reynolds, Patrick Higgins

**Affiliations:** ^1^ Department of Radiation Oncology University of Minnesota Med School‐Twin Cities Minneapolis MN USA

**Keywords:** surface dose, extrapolation method, ionization chamber, OSL, TLD, film

## Abstract

The purpose of this study was to review application of a consistent correction method for the solid state detectors, such as thermoluminescent dosimeters (chips (cTLD) and powder (pTLD)), optically stimulated detectors (both closed (OSL) and open (eOSL)), and radiochromic (EBT2) and radiographic (EDR2) films. In addition, to compare measured surface dose using an extrapolation ionization chamber (PTW 30‐360) with other parallel plate chambers RMI‐449 (Attix), Capintec PS‐033, PTW 30‐329 (Markus) and Memorial. Measurements of surface dose for 6 MV photons with parallel plate chambers were used to establish a baseline. cTLD, OSLs, EDR2, and EBT2 measurements were corrected using a method which involved irradiation of three dosimeter stacks, followed by linear extrapolation of individual dosimeter measurements to zero thickness. We determined the magnitude of correction for each detector and compared our results against an alternative correction method based on effective thickness. All uncorrected surface dose measurements exhibited overresponse, compared with the extrapolation chamber data, except for the Attix chamber. The closest match was obtained with the Attix chamber (−0.1%), followed by pTLD (0.5%), Capintec (4.5%), Memorial (7.3%), Markus (10%), cTLD (11.8%), eOSL (12.8%), EBT2 (14%), EDR2 (14.8%), and OSL (26%). Application of published ionization chamber corrections brought all the parallel plate results to within 1% of the extrapolation chamber. The extrapolation method corrected all solid‐state detector results to within 2% of baseline, except the OSLs. Extrapolation of dose using a simple three‐detector stack has been demonstrated to provide thickness corrections for cTLD, eOSLs, EBT2, and EDR2 which can then be used for surface dose measurements. Standard OSLs are not recommended for surface dose measurement. The effective thickness method suffers from the subjectivity inherent in the inclusion of measured percentage depth‐dose curves and is not recommended for these types of measurements.

PACS number: 87.56.‐v

## I. INTRODUCTION

Surface dose and skin dose are important aspects of external beam radiation therapy. Surface dose should be measured to within at least±5% accuracy for beam modeling. Skin doses are often critical delimiters of patient treatment, either being too low when superficial doses are needed or too high when they are not. Surface doses may be adequately measured with parallel‐plate ionization chambers, but there are a variety of devices used for *in vivo* skin measurements. For these we would also like to achieve clinical accuracy in the ±5% range.

Surface dose measurements are complicated by detector design and are subject to variable electron contamination conditions due to field size, photon energy, and beam modifiers. Accurate *in vivo* measurement requires correcting detector response for each inherent problem. Some of the typical detectors used for surface dose measurements (and corresponding pertinent references) are listed in Table 1. The gold standard for surface dose measurements is the extrapolation chamber,^1^, ^2^ but only a few institutions have access to these instruments. The next best detector for surface dose measurement is the Attix parallel‐plate chamber.^3^, ^4^, ^5^ The advantage of the Attix chamber is its primarily solid water construction and minimal overresponse due to side wall in‐scatter (<1%).^(4)^ Most other fixed separation parallel‐plate chambers are not constructed of solid water, but have been used for surface dose measurement with appropriate corrections for polarity, obliquity, and in‐scattering.^(2)^ The advantage of TLDs, OSLs, and films is their small size and tissue equivalence; therefore, they can be used as *in vivo* dosimeters where ion chambers cannot. A good agreement with Monte Carlo calculations was demonstrated with 0.1 mm TLD measurements done for a series of different beam angles and energies.^(6)^ The effect of chip thickness on TLD dose measurement has been reported.^(2)^

Linear extrapolation to zero depth using three TLDs has been used in a clinical setting for the assessment of the effect of different types of boluses on skin.^(5)^ In another study extrapolation using three different thickness TLDs showed a slight overestimation of surface dose demonstrating nonlinear TLD response versus TLD thickness.^7^, ^8^ This is not the case for TLD powder (pTLD), and a monolayer of powder can be used for surface dose measurements.^(2)^ Regarding OSLs, most reports concentrate on the investigation of OSL characteristics and their response at $d_{\text{ref}}$ or deeper.^(9)^ Only a few have reported the use of OSLs in the buildup region for skin dose measurements.^10^, ^11^

**Table 1:** Dosimeters used for estimation of surface dose

*Dosimeter*	*Detectors Use for Surface Dose Measurements*	*Refs*
PTW 30‐360	Gold standard for surface dose, but it cannot be utilized for *in vivo* measurements	1,2
Attix	Buildup dose within 1% for 6 MV photon beams, even in highly contaminated beams	3
	Less than 1% overresponse due to side scatter	4
Other parallel‐plate detectors	Large perturbation effects, up to 15% corrections are required for buildup regions	32
	Overestimation of the dose in the buildup regions due to in‐scattering of the secondary electrons from the side wall of the chamber	1,33
cTLD & pTLD	Shown to overestimate surface dose by up to factor of 2 due to the chip thickness	1
	TLD chips yielded surface doses that were 12% high	2
	Extrapolation method to zero thickness using 1‐3 different thickness TLDs reported good agreement with parallel‐plate results	7
	3% agreement with Monte Carlo calculations	6
	A monolayer of TLD powder was suggested for institutions lacking an extrapolation chamber for determining percent depth dose throughout the entire buildup region	2
OSL	Overestimates the surface dose due to intrinsic buildup	10
EDR2	Ready‐packed film overestimates surface dose by 10%	12
	Significant overresponse to low energy radiation by silver	13
	Extrapolation method using three stacked films showed that surface dose can be estimated to within±3% of parallel‐plate results	15,16
EBT2	Extrapolation method yielded agreement to within 2%‐3% of extrapolation chamber	19,20
	Skin dose correction for the effective point of measurement (EPM) is negligible for radiochromic film	
	In homogeneous conditions, EPM of EBT2 film can be considered equivalent to the clinical skin depth of 0.07 mm	21

The advantage of film over the afore‐mentioned detectors is that they produce an accurate high‐resolution, two‐dimensional map of surface dose. AAPM Task Group 69 report discusses some of the limitations and provides recommendations for the uses of radiographic films.^(12)^ Radiographic film is a relatively inexpensive alternative for two‐dimensional surface dose assessment, but care should be taken due to the chemical processing of the film and possible air‐pockets under the “ready‐pack” wrapper. Use of an extrapolation method with three stacked films showed that the percentage surface dose can be estimated to within±3% of parallel‐plate results.^(13)^ Radiochromic film, on the other hand, does not require any postprocessing and, therefore, has smaller dose uncertainty (3.3%).^(14)^ Similar to radiographic film, the extrapolation method has also been used for assessment of surface dose (2%–3% agreement with extrapolation chambers).^15^, ^16^ Thus, dose buildup due to the thickness of EBT2 and thickness and packaging of EDR2 films suggest the need for corrections to be made as for the point detectors.

Most treatment planning systems are usually not accurate estimators of surface dose unless very well‐modeled, using corrected surface dose data.^(11)^ Otherwise, models tend to overestimate surface dose.^(17)^ In addition, any beam modifying devices have a large impact on the actual, compared with modeled, surface dose. This problem is recognized in the AAPM report on quality assurance for clinical radiotherapy treatment planning, describing differences of up to 20% between TPS calculations and measurements.^(18)^ In this report we describe a consistent use of a linear extrapolation method for solid‐state detector thickness corrections, and show how to benchmark them for accuracy against ionization chamber measurements.

## II. MATERIALS AND METHODS

### A. Experimental setup, linear accelerator, and field arrangements

**Figure 1:**
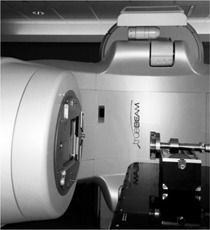
The experimental setup of surface dose measurement with extrapolation ion chamber PTW 30‐360.

Surface dose measurements were performed on a 6 MV X‐ray beam (Varian Clinac 2300, $10\times 10\;{\text{cm}}^{2}$ open field, SSD=100 cm; Varian Medical Systems, Palo Alto, CA). The parallel‐plate chambers were flush‐mounted in either custom‐built polystyrene phantoms (Capintec (Ramsey, NJ), Markus (PTW, Frieburg, Germany), and Memorial (CNMC Co., Nashville, TN), or a Solid Water phantom (Attix; RMI, Middleton, WI). The extrapolation chamber, due to its design, was not embedded in a phantom but was mounted horizontally on the couch so that solid water buildup slabs could be propped against it (Fig. 1). All of the solid‐state detectors were simply placed on a Solid Water phantom. In each case, measurements were made at the surface and dmax (1.7 cm).

Parallel‐plate chamber measurements were first intercompared with and without (published) corrections to help validate the PTW 30‐360 baseline measurements and to demonstrate the order of magnitude of these corrections in comparison with the solid‐state detectors.

**Figure 2:**
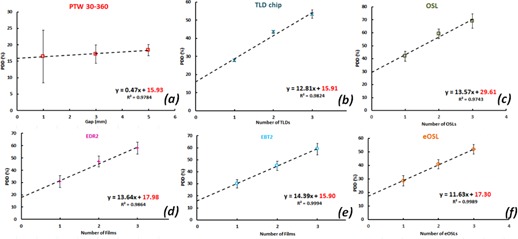
Measured data and extrapolated surface dose for each detector: (a) PTW 30‐360, (b) cTLD, (c) OSL, (d) EDR2, (e) EBT2, (f) eOSL.

The extrapolation technique consists of irradiating a stack of three detectors placed on top of a Solid Water phantom. Each detector has unit thickness in this simple method and was separately read out with the outermost dose associated with thickness 1, the center dose with thickness 2, and the dose to the detector in contact with the surface with thickness 3. These doses were then linearly fit against thickness number to obtain the surface dose (Figs. 2(b) to 2(f)). Surface doses were then normalized to single detector dose measurements at dmax to obtain relative surface doses for comparison with the baseline PTW 30‐360 measurements. This process was carried out for all of the solid‐state detectors: cTLD, OSLs, EDR2 film, and EBT2 radiochromic film. OSLs were tested in two configurations, nominally closed (OSL) and externally exposed (eOSL), to evaluate the effect of the enclosure.

### B. The PTW 30‐360 extrapolation chamber

An extrapolation chamber manufactured by Physikalisch‐Technische Werkstatten (PTW, model 30‐360) was used for baseline measurement of surface dose. The physical characteristics of this chamber were described earlier by Gerbi.^(3)^ The linac gantry was rotated to 270° (parallel to the couch), and the PTW 30‐360 chamber surface was aligned with the central axis of the beam with or without solid water buildup taped to it and set to 100 cm SSD for each measurement (Fig. 1). The chamber was connected via a coax cable to a Keithley model 602 electrometer (Keithley Instruments, Cleveland, OH). A separate, regulated power supply provided variable bias voltage, depending on plate separation. Polarity effects were included by averaging measurements at both polarities. Measurements were acquired for electrode separations of 5 mm, 3 mm, and 1 mm. Surface and buildup doses were evaluated by linear extrapolation of the charge collected to zero plate separation.

### C. Fixed separation parallel‐plate chambers

Fixed separation parallel‐plate chambers included an Attix Chamber RMI model 449, a Capintec PS‐033 thin‐window ion chamber, a Memorial Pipe Chamber with a circular (1 cm diameter) collecting electrode and a Victoreen/Nuclear Associates model 30‐329 chambermanufactured by PTW Frieburg (the Markus Chamber). For detailed physical characteristics of the Attix chamber and other fixed separation parallel‐plate chambers, the reader is referred to the earlier publications by Gerbi and Khan^(2)^ and Gerbi.^(3)^ All chambers were connected via a triaxial cable to a Keithley model 602 electrometer at −300V bias voltage.

### D. Radiographic EDR2 and radiochromic EBT2 films

Radiographic film Kodak EDR2 (Carestream Health, Rochester, NY) was chosen for surface dose measurement due to its wide availability, low cost, wide response range (0–400 cGy), and relative linearity. Surface and $d_{\text{ref}}$ films were processed sequentially in order to reduce the effect of dependent factors, including processing conditions (processing time, temperature, equipment, chemistry), density sampling (digitizer equipment and calibration), and exposure monitoring equipment. Each film was in the original pack, which consisted of the package envelope, paper separator, and unexposed film. Two hundred monitor units were delivered in a 6 MV $10\times 10\;{\text{cm}}^{2}$ fields at 100 cm SSD. All films were processed in a Kodak RP X‐Omat processor (F0038519) 24 hours after exposure. The processed films were then scanned using a VidarVXR‐12 visible light densitometer and analyzed using RIT113 imaging software using a measured calibration curve. Film doses were averaged from the central approximately equal to $4\;{\text{cm}}^{2}$ and normalized to the corresponding $d_{\text{ref}}$ measurements.

The choice of radiochromic film for surface dosimetry was made due its energy independence, tissue equivalency, high sensitivity, self‐development, and concise usage.^16^, ^19^, ^20^, ^21^ A sheet of film (of area $20.3\times 27.9\;{\text{cm}}^{2}$) was scanned with an Epson Perfection V700 Dual Lens system Photo Scanner (US Epson, Long Beach, CA) and cut into $4\times 5\;{\text{cm}}^{2}$ pieces and marked in order to distinguish the side of the polyester overlaminate layer. This side was positioned faceup for all measurements to ensure the active layer was located closer to the surface. The 30 μm thick active layer is positioned 75 μm from the surface of the film in this orientation, as opposed to 175 μm in the opposite side. In order to obtain precise optical densities, the irradiated films were left to self‐develop for 24 hours or longer, and the scanner was switched on 15 min prior to readout to allow for lamp warm‐up. Background optical densities were read prior to irradiation. In order to minimize the effect of sensitivity variation with position within the film, postirradiation optical density was averaged from three different points on each piece of film. The net optical density was defined by the subtraction of the background optical density from the exposed optical density. All data were analyzed in the red channel over the range 1–1000 cGy.

### E. Thermoluminescent detectors

TLD‐100 thermoluminescent detector chips ($3.1\times 3.1\times 0.38\;{\text{mm}}^{3}$) and powder from Harshaw/Filtrol Partnership (Louisville, KY) were used for relative surface dose measurements. Each chip was individually calibrated by repeat irradiation to a known dose, and read using a Harshaw Model 3000 reader (Thermo Fisher Scientific Inc., Waltham, MA) with nitrogen flow. By following this procedure and annealing the chips in a 400° oven for 1 hr between exposures, we can expect 3% measurement precision.^(22)^ A calibrated powder dispenser was used to regulate the amount of exposed powder (15 mg) to be read, which was then vibrated into an even, approximately monolayer, distribution on the planchette. The TLD chips and powder were read out at least 24 hrs after exposure to allow for background decay.^(23)^

### F. Optically stimulated detectors

InLight nanoDot OSLs (Landauer Inc., Glenwood, IL) are particularly attractive for surface dosimetry because they are reusable, thin, and easy to place on the patient's skin, and have been reported to provide accurate dosimetric results in other contexts. OSLs are plastic disks infused with 60% Aluminum Oxide ($\rho =3.95\;g/{\text{cm}}^{3}$) doped with Carbon (Al_2_O_3_:C) with dimensions of 3 mm radius and 0.2 mm thickness. Due to its sensitivity to visible light, the active area of the detector is encapsulated in a $1\times 1\times 0.185\;{\text{cm}}^{3}$ protective plastic box with a mass density of 1.03 g/cm^3^, having an effective point of measurement (EPM), which is the water‐equivalent depth from surface to half active layer, of 0.8 mm. Similar to TLDs, in order to avoid a short‐term signal decay period, the OSLs were read with an InLight MicroStar reader at least 30 min after exposure.^24^, ^25^ All OSLs were preexposed to 1 kGy, as suggested by Jursinic^(25)^ in order to better linearize further dose response. After irradiation and readout, nanoDots were optically bleached in a light source. OSL doses were read by applying the multidose calibration option in the InLight MicroStar reader. This setting switches between a low and high intensity LED‐beam depending on pretested dose level to give accurate high‐ and low‐dose readings, respectively.

## III. RESULTS

Table 2 displays surface dose measurements obtained with all of the detectors before any corrections were applied, including Velkley corrections^(30)^ for the ionization chambers and extrapolation corrections for the solid‐state detectors. The results are the means±standard deviations (SDs). Noticeably, all single detector surface dose measurements were overestimated, compared with the polarity‐averaged extrapolation chamber measurements, except for the Attix chamber. The absolute percent difference between actual and measured, but uncorrected, surface doses ranged from −0.1% (Attix chamber) to 26% (OSLs).

No corrections were required for the Attix chamber and the pTLD measurements. Surface doses measured with these detectors were 15.81±2.08 (Attix) and 16.40±3.01 (pTLD) in close agreement with the extrapolation chamber (15.93±2.10). Parallel‐plate measurements were corrected using the Gerbi and Khan method.^(2)^ The percent differences between the corrected measurements and the baseline were: 0.03%±2.97% (Capintec PS‐033), 0.94%±3.02% (Markus PTW 30‐329), and 0.23%±3.04% (Memorial pipe chamber).

The extrapolation correction method was applied to all solid‐state detectors readings and the results are illustrated in Fig. 2. Here the relative doses for each layer: top (number of detectors – 1), middle (number of detector – 2) and bottom (number of detectors – 3) are shown. The data were fitted with a linear relationship; the extrapolated surface dose is the y‐intercept. These results are summarized in Table 3. The first column lists the fitted slope for each detector, the second column the extrapolated percent surface dose, and the third column the residual difference with the baseline extrapolation chamber measurement. The percent difference in between the extrapolated doses for each detector and PTW 30–360 measurements are the following: −0.02±2.36 (cTLD), 1.37±4.14 (eOSL), 2.04±3.65 (EDR2), −0.03±4.54 (EBT2), 13.68±4.83 (OSL).

**Table 2:** Uncorrected percent surface dose using different dosimeters

*Ion Chambers Detectors*	*Measured Dose (%)*	*Solid State Detectors*	*Measured Dose (%)*
PTW 30‐360	15.93±2.10	EBT2	30.10±2.12
Attix	15.81±2.08	EDR2	30.69±3.71
Capintec	20.39±2.10	OSL	41.91±4.14
Markus	25.95±2.17	eOSL	28.70±3.45
Memorial	23.23±2.20	cTLD	27.72±3.05
		pTLD	16.40±3.01

**Table 3:** Dose correction factor (fitted slope) and extrapolated surface dose for all detectors

*Detectors*	*Slope (PDD(%)/#detectors) (%)*	*Extrapolated Dose (%)*	*Residual Difference (%)*
cTLD	12.81±0.66	15.91±1.08	−0.02±2.36
eOSL	11.63±2.07	17.30±3.57	1.37±4.14
EDR2	13.64±1.74	17.97±2.98	2.04±3.65
EBT2	14.39±2.35	15.90±4.02	−0.03±4.54
OSL	13.58±2.56	29.61±4.35	13.68±4.83

**Figure 3:**
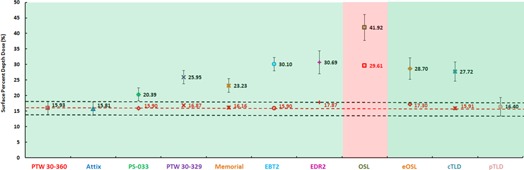
Percent surface dose measurements using different dosimeters. The dotted red line is the surface dose measured with the extrapolation chamber (PTW 30‐360); dotted black lines represent the SD in those measurements. Colored and red markers represent uncorrected and corrected surface doses, respectively, for each detector.

Finally, the uncorrected and corrected detector readings are summarized in Fig. 3. Error bars include measurement (SDs) and estimated systematic uncertainties. For the solid‐state detectors, the latter are in the range of 2%–3%.^20^, ^26^, ^27^, ^28^, ^29^ For all ionization chambers, we assume a systematic uncertainty of about 2%. Therefore, the total uncertainties for the ionization chambers were estimated as: PTW 30–360 − 2.10%, Attix − 2.08%, Capintec − 2.10%, Markus − 2.17% and Memorial − 2.20%, and for the solid‐state detectors as: EBT2 − 2.12%, EDR2 − 3.71%, OSL − 4.14%, eOSL − 3.45%, cTLD − 3.05%, and pTLD − 3.01%. Dotted lines correspond to the estimated SD (±2.10%) in the extrapolation chamber measurements.

## IV. DISCUSSION

The extrapolation chamber (PTW 30‐360) measurements were combined with the other corrected parallel‐plate chamber measurements for the establishment of the baseline surface dose. The Gerbi and Khan^(2)^ correction method is an extension of the Velkley et al. method,^(30)^ which includes the effect of the collector edge‐sidewall distance of the chamber, enabling correction of the chamber response as a function of depth and energy, although this correction does not take into account the specific size of the collector and electrode. This correction method worked well for all of the parallel‐plate chambers, bringing all corrected surface doses to within 1% of the extrapolation chamber result.

Overestimation of surface doses measured with the solid‐state detectors is assumed to mostly be due to the detectors' physical thickness. Surface dose overestimation by more than 10% has been reported for TLD chips by both Gerbi and Khan^(2)^ and Kron et al.,^(7)^ and for both EDR2^(12)^ and EBT2.^(15)^

The common extrapolation method we have employed has corrected all of the solid‐state detector measurements to within 2.10% of the extrapolation chamber reading, except for OSLs. Previously reported extrapolated results for EDR2, EBT2, and TLDs were also in good agreement with our results.^7^, ^13^, ^15^, ^16^ The extrapolation method, using a linear fit, is demonstrated in Fig. 2 and includes the extrapolation chamber baseline measurements at 1, 3, and 5 mm plate separation (Fig. 2(a)). The smallest separation of 1 mm of extrapolation chamber resulted in a larger error bar due to the noise associated with the low signal and much larger polarity correction. The equation of the linear trend line, drawn through the relative doses for each thickness, was acquired. We can use the fit results to allow us to correct *in vivo* surface dose measurements by simply subtracting from them the product of the slope times the number of detectors (usually one). The failure of the extrapolation method in the case of OSLs is explained by the presence of the protecting case. Open OSLs exhibited a much more predictable response, as shown in Fig. 3. Extrapolation based on number of stacked detectors for the standard OSL closed configuration is inadequate and should not be relied upon. In addition, the extrapolation method was developed for ideal geometry, with the surface of the phantom perpendicular to the central axis of the beam. Therefore, added measurement uncertainties due to obliquity and other *in vivo* setup issues decrease the overall accuracy somewhat, but we think it is better than the EPM method. Although we have not looked at other energies, we anticipate similar success.

Another approach to compensate for this is to determine the effective point of measurement (EPM) for each detector and apply an appropriate percent depth‐dose correction. For instance, the EPM for a Landauer OSL was determined by Zhuang and Olch^(11)^ to be $D_{\text{OSL}}=0.8\;\text{mm}$, and 0.07 cm^2^/gm by Reft.^(31)^ The thickness of TLD100 was 0.38 mm ($\rho =2.64\;g/{\text{cm}}^{3}$); therefore, with the assumption that the EPM is located at the midpoint of the TLD, then $D_{\text{TLD}}=0.5\;\text{mm}$ of water. The EPM for EBT2 and EDR2 were reported previously: $D_{\text{EBT}2}=0.07\;\text{mm}$ ^(20)^ and $D_{\text{EDR}2}=0.38\;\text{mm}$.^(13)^ However, the steep gradient in PDD (15.9% to 44.7% within the first millimeter in a 6 MV photon beam for a field size of $10\times 10\;{\text{cm}}^{2}$) makes any effective depth correction extremely sensitive. In contrast to the extrapolation method, when we applied the EPM approach we found it predicted higher surface doses for OSL, EDR2, and EBT2, with differences between baseline and measurement of 5.8%±4.9%,4.4%±4.2%, and 9.8%±2.1%, respectively, and 1.8%±2.6% lower for cTLD. Except for EBT2, these results for the EPM method fall within our±5% criteria but are very dependent on the accuracy of the baseline depth‐dose curve, whereas the extrapolation method is not. Consequently, the EPM method may be the less subjective choice of the two methods.

## V. CONCLUSIONS

We have demonstrated that baseline surface doses can be measured with a variety of parallel‐plate ionization chambers and used to evaluate the accuracy of corrected solid‐state detector measurements. A simple extrapolation method can provide nonsubjective thickness corrections for a number of detectors such as cTLD, eOSLs, EBT2, and EDR2. Standard packaged OSLs were found to not be correctable using this method.

We found that, except for OSLs, single detector measurements all exhibited approximately similar overresponse of about 13%, consistent with all of them having close to the same effective thickness. Inclusion of this correction successfully resulted in surface dose measurement accuracy of within about 2%–3%, which should enable *in vivo* dosimetry with these solid‐state detectors to be within a clinical target goal of ±5%. The EPM method suffers from the subjectivity inherent in the inclusion of measured percentage depth‐dose curves and is not recommended for these types of measurements. This work demonstrates the consistency of thickness extrapolation for general surface dose measurement.
